# Manual for nursing consultation with people living with Diabetes Mellitus: implementation science

**DOI:** 10.1590/0034-7167-2024-0179

**Published:** 2025-12-08

**Authors:** Simone Orth, Leila Zanatta, Elisangela Argenta Zanatta, Vilma Beltrame, Elizabeth Teixeira, Edlamar Kátia Adamy

**Affiliations:** I Universidade do Estado de Santa Catarina. Chapecó, Santa Catarina, Brazil; II Universidade do Oeste de Santa Catarina. Joaçaba, Santa Catarina, Brazil; III Universidade Estadual do Pará. Belém, Pará, Brazil

**Keywords:** Diabetes *Mellitus*, Office Nursing, Technology, Validation Study, Implementation Science., Diabetes *Mellitus*, Enfermería de Consulta, Tecnología, Estudio de Validación, Ciencia de la Implementación.

## Abstract

**Objectives:**

to describe the process of content validity, implementation and assessment of use of a manual for nursing consultation for people living with Diabetes *Mellitus*.

**Methods:**

methodological research, with an interface with principles of implementation science developed in three stages: content validity; implementation; and use assessment.

**Results:**

content was assessed by seven experts, obtaining a general Content Validity Index of 0.80 and a Content Validity Ratio of 0.72. The manual, after adjustments based on recommendations received, was entitled “Consulta do Enfermeiro às pessoas que convivem com Diabetes *Mellitus*”, organized in 67 pages, containing five chapters. Implementation took place in 24 municipalities in the west of Santa Catarina over a period of four months, and 43 nurses participated. User experience assessment was carried out with six nurses, and analysis resulted in three categories.

**Final Considerations:**

the manual met the proposed assessment parameters, revealing evidence and appropriating it for use.

## RESUMEN

**Objetivos:**

describir el proceso de validación de contenido, implementación y evaluación del uso de un manual para la consulta de enfermería con personas que viven con Diabetes *Mellitus*.

**Métodos:**

investigación metodológica, con interfaz con los principios de la ciencia de la implementación desarrollada en tres etapas: validación de contenido; implementación; y evaluación de uso.

**Resultados:**

el contenido fue evaluado por siete expertos, obteniendo un Índice de Validez de Contenido General de 0,80 y un Content Validity Ratio de 0,72. El manual, luego de ajustes basados en las recomendaciones recibidas, se tituló “Consulta do Enfermeiro às pessoas que convivem com Diabetes *Mellitus*”, organizado en 67 páginas, conteniendo cinco capítulos. La implementación se realizó en 24 municipios del oeste de Santa Catarina durante cuatro meses y participaron 43 enfermeras. Se realizó una evaluación de la experiencia del usuario con seis enfermeras y a partir del análisis se estructuraron tres categorías.

**Consideraciones Finales:**

el manual cumplió con los parámetros de evaluación propuestos, revelando evidencia y haciéndolo apto para su uso.

## INTRODUCTION

Diabetes Mellitus (DM) is a chronic disease in which the body does not produce insulin or cannot properly use the insulin it produces. Classified as a chronic non-communicable disease, prevalent throughout the world, it is considered a Primary Health Care (PHC)-sensitive condition^(1)^.

In 2021, approximately 536 million people were living with DM worldwide, and the projection for 2045 is 783 million, an increase of approximately 46%. Comparing South America and Central America, in 2021, there were approximately 32 million individuals affected by the disease, with a projection for 2045 of approximately 49 million, an increase of 53%. Meanwhile, in Brazil, in 2021, there were 15 million individuals living with DM, with an expected increase of 47% for 2045, equivalent to approximately 23 million inhabitants affected by the disease^(2)^.

Professionals working in PHC have a fundamental role in improving these indicators; to do so, they need to raise awareness and assist the population in the process of recognizing the pathology, adopting positive attitudes in preventing complications^(3)^. Creating bonds, analyzing the profile of the population enrolled in the area, managing symptoms and complications, and joint planning between healthcare professionals, patients and families are essential strategies. Therefore, providing support and support to people living with DM allows for greater control over the disease and its long-term effects^(4)^.

Thus, with the increasing prevalence of DM and its associated complications, especially in the Brazilian context, the pathology affects millions of individuals and is projected to continue to grow in the coming decades^(2)^. DM represents a significant burden on the health system, being one of the main causes of morbidity and mortality in the country and worldwide, with a direct impact on affected individuals’ quality of life. In this regard, PHC plays a fundamental role in disease prevention and management, especially when combined with nurses’ ability to provide adequate care, promote self-care and manage complications^(3)^.

In this context, nurses have the ability to improve this population’s quality of life and cope with the disease through nursing consultation (NC). According to Resolution 736 of 2024 of the Federal Nursing Council (In Portuguese, *Conselho Federal de Enfermagem* - COFEN)^(5)^, consultation must be organized and recorded according to the nursing process (NP) stages, and may be mediated by the application of validated technologies.

The assessment and implementation of these technologies are carried out by implementation science, which associates practices that are based on evidence, taking into account factors such as the setting, population characteristics and implementation characteristics that can influence their implementation and maintenance in the context in which they are inserted^(6)^.

Therefore, time and financial situation are essential conditions for a project to be developed in its entirety by a researcher. In addition to the complexity of each phase, the schedule is sometimes the greatest adversary of the process. Therefore, it is important to adopt research continuity projects, in order to cover all phases, ensuring that technological solutions are actively implemented and assessed^(7)^.

The manual for NC with people living with DM was developed by an undergraduate nursing student, a scientific initiation scholarship holder from a public university located in western Santa Catarina, in two phases: in the first, a narrative literature review was carried out; and in the second, construction took place. This article concerns a follow-up study that validated and assessed the experience of using this manual, considered an instructional teaching material technology. The developed manual was able to optimize professionals’ approach in PHC, improving treatment adherence, glycemic control and the quality of life of patients living with DM. By integrating evidence-based practices and validated technologies into nursing care, it will be possible to reduce complications and promote better health outcomes for this population.

Thus, to ensure manual quality and applicability, content validity was carried out with the participation of experts in the field, guaranteeing its relevance and suitability. The implementation followed the Consolidated Framework for Implementation Research (CFIR) principles, allowing a structured approach for its practical application. In addition, a use assessment was conducted to verify material effectiveness and acceptance. The data were analyzed using the Content Validity Index (CVI), Content Validity Ratio (CVR), Kappa coefficient for inter-rater agreement and Bardin content analysis, ensuring methodological rigor and reliability of results.

## OBJECTIVES

To describe the process of content validity, implementation and assessment of use of a NC manual for people living with DM.

## METHODS

### Ethical aspects

This study is linked to the research macroproject entitled “*Desenvolvimento de tecnologias para a Consulta do Enfermeiro nas Redes de Atenção à Saúde*”, which was approved by the *Universidade do Estado de Santa Catarina* Research Ethics Committee, under Protocol 5,047,628 and Certificate of Presentation for Ethical Consideration 50165621.2.0000.0118.

### Study design, period and location

This is a methodological research^(8)^ of an exploratory and descriptive nature, with an interface with principles of implementation science^(9)^, developed in three adapted stages: content validity; implementation; and use assessment. It was carried out from January to December 2023, characterizing itself as a continuity project^(7)^.

### Sample; inclusion and exclusion criteria

In the first phase of the study, a list of potential experts for content validity was drawn up, identified from the *Curriculum Lattes* registered on the Brazilian National Council for Scientific and Technological Development Platform, which met at least two of the following inclusion criteria^(10)^: being a specialist in DM; having clinical-care practice with the target audience of the study for at least three years; having published work in a journal and/or event on DM; having published works in journals and/or events on the construction and validity of care-educational technologies in DM; being a Brazilian Diabetes Society member. Exclusion criteria were sending the responses after the specified deadline.

For the implementation and validity stages, the following inclusion criteria were established: being a nurse and working in PHC; caring for people living with DM; working in the western region of Santa Catarina. Exclusion criteria were being on vacation or leave or not responding to the questionnaire within the established deadline.

### Study protocol

In the first phase, six to 20 experts were considered. A minimum of six experts is recommended^(11)^ to ensure a significant consensus, and a maximum of 20 experts, which contributes to the diversity of opinions and reduction of individual biases, increasing the robustness of the findings. Validity according to the model gives the product greater credibility, based on scientific evidence, highlighting the importance of the participation of experts in the subject^(12,13)^.

Upon acceptance, the Informed Consent Form and instructions for completing the content validity instrument were forwarded, including the option to suggest improvements for each item assessed,as well as the manual as a whole. The manual was sent via email in January 2023, with a link to access the instrument via Google Forms^®^. Experts had 30 days to provide feedback, but due to the vacation period, the deadline was extended for another 30 days, concluding this stage of the study in March 2023.

The content validity instrument was adapted^(14)^ and organized into three chunks (1) expert characterization; 2) instructions on completing the instrument; 3) questions for content validity), which assess the manual objectives, structure and presentation/relevance, with a score assigned to each item assessed, according to the degree of agreement.

In the third chunk, the objectives item was composed of five subitems for analysis, 12 of which focused on structure/presentation and five on relevance, totaling 22 subitems analyzed. Each subitem was assessed using the Likert scale, assigning scores from 1 to 4, as follows: 1 - inadequate; 2 - partially adequate; 3 - adequate; and 4 - totally adequate. When assigned a grade of “1” or “2”, the instrument provided space for the description of the reason or suggestion related to the item assessed.

In the second phase, in light of implementation science, the CFIR principles were used to implement and assess user experience. Implementation science allows organizing implementation strategies for the process of inserting measures and demands within the desired setting, allowing a critical and comprehensive analysis of the entire process^(15)^. The following CFIR domains were used: intervention characteristics; inner setting; and characteristics of individuals^(9)^. It was carried out with nursing assistants who work in the PHC of the 24 municipalities that make up the Western Health Region of Santa Catarina, which has 174 nurses working in PHC^(16)^. During this phase, three moments of dissemination and invitation were carried out: 1) sending an email to the State Department of Health management and to the technical nurse in charge of each municipality from May to July 2023; 2) holding an online meeting with nurses from the municipalities on July 6, 2023, lasting 30 minutes; 3) holding an in-person meeting at the health management of the Western Health Region of Santa Catarina, on August 17 of the same year. For the municipalities that were not represented on any of the occasions, contact was established via email from May to August 2023. A total of 43 nurses from the region agreed to participate.

In the third phase, after four months of implementing the manual, in December 2023, an instrument to assess user experience was sent to the same 43 nurses who agreed to participate in the study. Thus, nurses received an instrument consisting of 16 open-ended questions, with the objective of exploring how the manual was perceived by them in relation to the different domains, considering aspects such as technology acceptance, feasibility and applicability in the care of patients living with DM. The questions were formulated to collect qualitative data that allowed us to understand nurses’ experience in daily practice, which was facilitated by the use of Google Forms^®^ as a data collection platform. Nurses had 30 days to answer the questionnaire, which provided enough time for reflection and detailing of the answers. When using the CFIR, the planning and implementation of approaches to participants follow constructs, but the choice that best meets the study’s characteristics and needs is allowed, and the use of all constructs is not mandatory^(9)^.

### Data analysis and statistics

For content validity, the results of experts’ assessment were organized in Microsoft Office Excel 2013^®^ and analyzed according to descriptive statistics, with a view to obtaining the CVI, which allows the measurement of the proportion of experts who agree with the data presented in the manual, allowing validity as a whole or of each item individually. For the manual to be considered validated, a general CVI greater than or equal to 0.80 was considered. To calculate the CVI, the following formula was used: “CVI = No. of responses '3' and '4' ÷ Total No. of responses”^(13)^. Furthermore, the data were assessed according to the CVR, which consists of a measure that assesses content validity based on experts’ opinion, who classify the items as essential, useful or non-essential. The CVR is calculated by the formula CVR=[2N-(n_e_/2N)] / (N/2), where n_e_ is the number of experts who consider the item essential and N is the total number of experts. The CVR value ranges from -1 to 1, with values greater than 0.59 indicating good content validity. Positive values mean that the item is considered essential by experts^(17)^. The data were analyzed using the Kappa coefficient (*k*), recommended in data analysis in the area of health research, with the aim of measuring the level of agreement among experts. In other words, the degree of agreement, reliability and accuracy in classification were assessed^(18)^.

To assess user experience, participants’ responses were transcribed in full, using categorical content analysis, operationalized in pre-analysis, categorization, and results processing and interpretation phases^(19)^.

## RESULTS

Seven experts participated in the first phase (content validity). In relation to characterization, it was found that: in terms of age, they were between 35 and 53 years old; in terms of sex, five were female and two were male; in terms of training area, all were in nursing, with a doctoral degree and training time of 16 to 32 years. Concerning manual assessment, it was carried out in a single round, obtaining CVI by item and general CVI (Table 1). Regarding the analysis by the k coefficient, in which agreement among experts’ responses was verified, a statistically significant value was obtained (p-value = 0.0014).

**Table 1:** Content validity with experts, Chapecó, Santa Catarina, Brazil, 2023

Item	TA	A	PA	I	CVI	CVR
**OBJECTIVES**						
1. Information/content is consistent with the daily needs of nurses who care for people living with DM.	2	4	1	0	0.86	0.76
2. Manual content allows nurses to understand DM.	3	3	1	0	0.86	0.76
3. Content invites and/or instigates changes in nurses’ behavior and attitude.	2	2	2	1	0.57	0.14
4. Content encourages manual use in practice/performance.	2	3	1	1	0.71	0.43
5. Manual content helps to clarify possible doubts about DM.	2	3	2	0	0.71	0.43
**STRUCTURE/PRESENTATION**						
6. Content is presented in language appropriate for nursing professionals.	4	2	1	0	0.86	0.76
7. Content is presented in interactive language, capable of capturing nurses’ attention.	3	3	1	0	0.86	0.76
8. Content follows a logical sequence.	4	2	1	0	0.86	0.76
9. Manual content includes information relevant to the nursing consultation stages.	5	1	1	0	0.86	0.76
10. The manual is appropriate for guiding nurses’ clinical and critical reasoning.	2	4	1	0	0.86	0.76
11. Content on the nursing consultation stages includes all relevant information.	4	1	2	0	0.71	0.43
12. The information presented is scientific.	4	0	3	0	0.57	0.14
13. Information is well structured in terms of agreement and spelling.	2	3	2	0	0.71	0.43
14. Information in the manual is objective and clear.	2	3	2	0	0.71	0.43
15. Information is enlightening.	2	4	1	0	0.86	0.76
16. Information is necessary and relevant.	5	2	0	0	1.00	1.00
17. Text size and font in the manual are appropriate.	3	3	1	0	0.86	0.76
**RELEVANCE**						
18. Manual content encourages learning.	4	3	0	0	1.00	1.00
19. The manual contributes to knowledge in the area.	5	2	0	0	1.00	1.00
20. Manual content sparks interest in the topic.	4	2	1	0	0.86	0.76
21. The manual addresses the subjects necessary for nurses to know.	5	1	1	0	0.86	0.76
22. The manual is suitable for use by nurses in consultations with patients living with DM.	2	2	2	1	0.57	0.14
Total					0.80	0.72

*DM - Diabetes Mellitus; TA - Totally Adequate; A - Adequate; PA - Partially Adequate; I - Inadequate; CVI - Content Validity Index; CVR - Content Validity Ratio.*

Regarding the suggestions received, 11 of recommendations for adjustments and modifications were accepted (Chart 1), with emphasis being placed, in the manual review, on items that did not obtain an CVI equal to or greater than 0.80 and/or a CVR equal to or greater than 0.59.

**Chart 1:** Expert comments/suggestions, Chapecó, Santa Catarina, Brazil, 2023

Item	Expert comments/suggestions	Judgment
1	Review the medications and use the Ministry of Health’s proposal regarding the medications available in the SUS network.	Accepted.
2	Very extensive manual for nurses to use in their daily lives.	Not accepted. A manual is characterized as an effective communication material that systematically and organizedly gathers important information and data on the subject, with the aim of bringing together a greater number of people, presenting essential knowledge.
3	Suggest further reading of other materials already published.	Accepted.
4	Add the hyperosmolar hyperglycemic state.	Accepted.
5	Include procedures (such as bariatric surgery) to treat the disease.	Not accepted, since it was not the focus of the study, in addition to the fact that the Brazilian Health System only offers surgery for the treatment of obesity, according to Ministry of Health Ordinance No. 424/2013.
6	Reordering of numbers and images.	Accepted.
7	Reordering of contents.	Accepted.
8	Replace the word “summary” with “presentation”.	Accepted.
9	Use the avatar on a larger number of pages.	Accepted.
10	Settings in the data collection instrument.	Accepted.
11	I recommend outlining the care that the client should take in case of hypoglycemia and which values ​​are considered low.	Accepted.
12	I recommend mentioning the Association of Diabetes Care & Education Specialists’ seven diabetes self-care behaviors.	Accepted.
13	Use citations throughout the text of updated guidelines and information from organizations that are directly related to diabetes care.	Accepted.
14	Inclusion of a chapter explaining how the manual was constructed.	Not accepted. The methodological process for preparing the manual is described in a Final Course Paper available in the university’s virtual library.
15	Follow the international consensus on foot and leg ulcers and their screening.	Not accepted. Specialist societies in the area use the term diabetic foot, such as the Brazilian Diabetes Society on its website, which describes assessments and care for diabetic feet.

**Figure 1:**
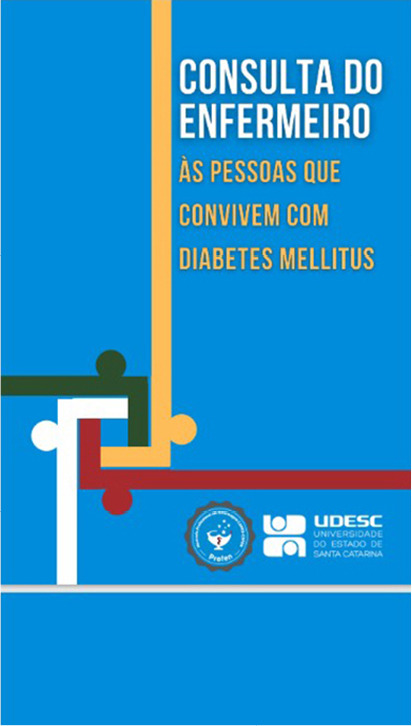
“*Consulta do Enfermeiro às pessoas que convivem com Diabetes Mellitus*” manual cover, Chapecó, Santa Catarina, Brazil, 2023

The manual was entitled “*Consulta do Enfermeiro às pessoas que convivem com Diabetes Mellitus*”. It is organized into 67 pages and five chapters: definitions and explanations about DM; classifications; non-pharmacological and pharmacological treatment; main complications arising from it; NC operationalization (Figure 1). The manual was registered with the International Standard Book Number by the Brazilian Book Chamber. It is available in full and free of charge on the Cofenplay platform.

The second phase involved 43 nurses working in 24 municipalities. The third phase involved six nurses from different municipalities who returned the instrument. Based on the analysis and the constructs adopted, three categories were structured: 1 - Intervention characteristics (adjustments to the setting that were implemented); 2 - Inner setting (involving structural, political and cultural context); 3 - Characteristics of indidivuals (the power of choice that influences decisions)^(9)^.

### Intervention characteristics

The manual contributes to the NP execution, guiding the implementation of the stages of this process. Its logical and sequential organization was highlighted for facilitating the identification of nursing diagnoses and interventions by nurses.

*Interesting, simple, easy to handle, educational instrument, it helped a lot to put together a consultation instrument.* (N1)

*It is very well organized, in a logical sequence, and helps us make diagnoses and interventions.* (N6)

The manual was described as interesting, simple and easy to handle, suggesting accessibility to nurses. Its practicality and relevance of information enhance its use. Therefore, the manual is a valuable tool that combines ease of use, practical relevance and effective support for performing the NP.

The importance of a manual with theoretical and scientific evidence to systematize consultation is undeniable, as it serves as a didactic guide to perform the NP, providing a solid basis for clinical decision-making. Furthermore, it supports the nurse technically and scientifically, ensuring safety and confidence in the performance of functions.

*More attention to the general physical examination and planning of strategic actions to prevent worsening of the disease.* (N2)

*Having theoretical support helps a lot in the consultation process* [...] *the manual is didactic and exemplifies the nursing process well.* (N4)

The statements presented highlight the effectiveness and convenience of the manual presented in digital format. Praise was given to its quality, suggesting only periodic updates to keep up with changes, which is a common and necessary practice in the digital age. The manual ease of access and use is emphasized, reinforcing the importance of accessibility in nurses’ user experience.

*As it is a digital tool, it makes things easier since we have access to a computer and the internet.* (N1)

*The manual is very good. I don’t think I would change anything, it’s on my computer’s desktop, easy to use at any time.* [...] *at first, I’m using it the way it is.* (N4)

Nurses highlight the ease provided by the digital tool, which suggests that their needs and expectations are met.

### Inner setting

Barriers to appropriate care for patients with DM are multifaceted and complex. One significant barrier is patients’ preference to see only their team physician due to medication prescription. This can limit patients’ access to appropriate guidance and care.

*Especially the time, the patient is always in a hurry and the demand on the unit is high.* (N4)

*Number of professionals working makes the everyday busy, sometimes making it difficult to use the manual regularly. Lack of some testing equipment and other proposed guidelines.* (N5)

Another barrier is the lack of support professionals, such as nutritionists and physical educators, who could provide valuable guidance to those living with DM. In addition, the high demand for daily nursing care and limited time are significant obstacles. Patients are often in a hurry, and the health care unit is very busy, which makes it difficult to conduct comprehensive assessments and regularly use the manual.

The manual is recognized as an informative resource that promotes reflection on the care provided, guiding the planning of nurses’ actions. However, it is valued for its ability to broaden nurses’ view of patient care, encouraging a more holistic assessment that goes beyond clinical indicators and considers patients’ family context.

*The manual provides information that makes us reflect on the care provided and also guided to people with DM, consequently changing our attitudes in relation to the planning of actions. Practicality, nursing consultation operationalization improvement.* (N2)

*It has allowed me to broaden my perspective on patient care, which is not just the level of glycated hemoglobin that I have to assess, but the entire person and their family situation. It guides me, I no longer ask vague questions.* (N4)

The assessments highlight the importance of comprehensive and personalized patient care, emphasizing the need for continuous attention and care. NC is seen as an essential tool for improving patient care.

Despite the difficulties mentioned, nurses experience feelings of positive change. Optimism and a sense of change are evident in the following statements:

[...] *whenever possible, read the manual to support daily work. I am adapting to using it.* [...] *I would recommend the manual to other nursing colleagues. I have already sent it.* (N3)

*To have standardized service, but in a unique way. The manual is very complete and helps in decision-making.* (N5)

They highlighted the manual’s importance and usefulness for nurses, recommending it to their colleagues and highlighting the lack of similar resources in their municipality and the frequency with which they serve this specific public.

The statements show the manual effectiveness in standardizing care, while allowing a unique approach for each case, and its contribution to the NP implementation and decision-making. They also emphasize the importance of familiarizing oneself with the manual and using it as a support resource in daily life, suggesting that it is not only a useful tool, but an instructional teaching material that contributes to qualifying nursing practice.

### Characteristics of individuals

The manual was considered a valuable instructional teaching material that qualifies the NC, considering its comprehensiveness, with complete content on DM, including strategies for patient care and a section for interview/data collection for the initial patient assessment.

*It is possible to carry out nursing consultation with all the steps in a clear and safe manner.* (N1)

*He goes above and beyond the minimum we should do for our patients. Sometimes, with the rush of everyday life, and without any material to guide us during consultation, we don’t even do the minimum.* (N6)

Nurses considered the need for other manuals that address other types of care, and highlighted the manual usefulness in daily nursing practice.

The lack of support from the State Health Department and nurses’ individual initiative in seeking improvements for the NP emerge. Although management supports the NC in general, the choice of the material to be adopted is at professionals’ discretion.

*In my municipality, it is nurses’ own initiative to improve the entire process.* (N4)

*For the specific use of the manual, no, but management supports nursing consultations in general, leaving it up to professionals to decide which support and material they prefer to use.* (N5)

This reinforces the idea that it is usually the nurses themselves who make the process of implementing nursing actions come to fruition. It suggests the need for greater institutional support and resources for nurses in order to improve the effectiveness and efficiency of patient care.

## DISCUSSION

Carrying out ongoing work allows research to be carried out at all stages, enabling products to be implemented in nursing practice, thus contributing to an improvement in praxis, with scientific theoretical foundation, and positive impacts on patients^(7)^.

Content validity allows for material improvement and technology qualification. The manual, in its final version, met the proposed assessment parameters, which proved that content is appropriate and valid, and can be reproduced in the scientific community, since it obtained a general CVI of 0.80. This index was similar to those obtained in other methodological studies of technology creation with content validity that used CVI for assessment by experts, such as “*O Manual de Cuidados de Idosos após Cirurgia Cerebral*”, which obtained a general CVI of 0.80^(20)^, and the work “*Construção e Validação de um Manual de Detecção do Pé Diabético para Atenção Primária*”, which obtained a general CVI of 0.85^(21)^.

As for scientificity, expert judges who assessed the content recommended the inclusion of additional data, which were incorporated into Chart 1, in items 11, 12 and 13. These suggestions were considered relevant and contributed to increasing the manual’s scientific basis. It is worth noting that these were the only suggestions made to improve the material’s scientific aspect.

Although handheld technologies may not be noticeable to patients, their use by professionals has become a significant resource for improving consultation and communication with patients. Therefore, they have an impact on the health sector, whether in primary care or at other levels of care^(22)^.

The technology used for consultation must be easy to understand and effective, being intuitive to obtain better results. These technologies emerge as facilitators of professionals’ work process^(23,24)^.

The manual complies with the Federal Nursing Council legislation^(5)^, which guides the NP implementation in all environments that have nursing care. This process must be systematized and organized according to the sector’s specific needs. This type of technology is inherent to the NC improvement, providing autonomy to nurses.

Thus, nurses’ autonomy is intrinsically linked to technologies, which serve as technical support and ethical guarantee in their professional practice. These technologies help to standardize care, encouraging decision-making and the use of standardized terminologies^(25)^. Encouraging nurses to use these technologies is essential to improve quality of care, combined with safety and training, which amplifies the care provided^(26)^.

Although NC has been around for many decades and is regulated by law, it still faces obstacles to its implementation. The practice during patient care is often neglected or carried out in a fragmented manner^(27)^. As the study shows, with the manual implementation, nurses are reflecting on NC and how it can improve patient care.

The manual soon received positive feedback from nurses, indicating that professionals’ perception of the usefulness, complexity, and adaptability of the intervention can influence the manual implementation. A study that used the CFIR in a health care manual indicated that professionals’ positive perception of the intervention and its ease of use are key reasons for the intervention adoption and sustainability^(28)^.

Nurses understand the relevance of using NP to improve care, but highlights several adversities, including excessive workload, shortage of human resources and lack of training^(29)^. There are several difficulties mentioned, including the need to lighten staff workload, with the aim of providing time and quality in care for individuals^(30)^.

Barriers such as excessive workload, shortage of human resources and lack of training that affect the NC implementation and manual use are directly related to the inner setting domain in the CFIR, which considers how the organizational structure and culture affect the implementation of a new intervention. In an international study, the CFIR was used to assess the implementation of a care manual for patients with diabetes, and it was identified that the availability of human resources and the continuous training of professionals were fundamental for the intervention sustainability^(31)^.

Therefore, professionals perform several functions, which include both management and care aspects. In other words, in addition to providing care to patients, they also organize the health unit, supervise the team and guide community health workers. Therefore, the time reported by nurses often becomes an obstacle due to the large number of tasks, and the time available for direct patient care ends up becoming increasingly limited^(32)^. In this context, nurses play a role in (re)organizing the work environment, facing a variety of challenges, whether in work dynamics, people management or local needs. The need for constant strategies and adaptations is deeply linked to the care process^(33)^.

NC promotes nurses’ autonomy, establishing a connection with patients, and encourages planning and organization of work. Nurses are essential professionals for strengthening PHC, offering assistance at all stages of life^(34)^.

The desire to involve patients in care, maximize the use of time and promote the NP implementation motivates nurses to seek and adopt tools that assist in consultation. In this way, they can offer effective, standardized and high-quality care^(26)^. Therefore, it is essential to encourage nurses’ autonomy through the use of technical-scientific support tools, as this promotes the empowerment of professionals in patient care^(35)^.

Identifying and meeting patients’ genuine needs, while avoiding unnecessary interventions, helps reduce unnecessary demand, allowing individuals with real needs to be assisted in a calm and efficient manner. In other words, the constant reassessment of actions and their consequences enables effective management of the real demand that exists in primary care^(36)^.

Therefore, the dyad NC and technologies in the nursing process are important points in patient care, becoming inherent to the care process. The technology used in NC allows data collection and its treatment coherently, resulting in excellent professional practice^(23)^.

Another study that used a framework to assess the implementation of health interventions reported that when technologies and work processes are adapted correctly and professionals feel supported and empowered, interventions have a positive impact on patient care and health outcomes. In the study, the impact of the manual is clearly visible in the quality of care provided and in the strengthening of nurse autonomy, which is a reflection of positive results indicated by the CFIR^(37)^.

Encouraging these professionals to use tools and provide them with notable returns in their practice will fuel the ambition for more positive results. The need for joint planning with management to incorporate NP into PHC is essential to improving care for the population^(29)^.

### Study limitations

The limitations found in this study were the short period between the implementation of strategies for using the manual in PHC and the subsequent user experience assessment. Therefore, it is suggested that future studies consider a longer period of time to allow a more accurate assessment of the strategies implemented.

### Contributions to nursing, health or public policy

The manual is available for use by nurses, aiming to enhance practices aimed at assisting the population living with DM in PHC and, thus, strengthen nurses’ work process, contributing to this population’s quality of life. The manual is applicable in different teaching settings, including undergraduate, graduate, residency and also in continuing or permanent education activities.

## FINAL CONSIDERATIONS

The manual met the proposed assessment parameters, revealing evidence that it is appropriate for use during NC. The manual allows quick and easy access to information, both for use in PHC and in nuse training/qualification, and contains illustrations that facilitate the handling and understanding of content by nurses. The manual use provides safety in professionals’ actions, but it is necessary to attract the target audience in order to demystify the idea that the care of people living with DM is limited to the clinical-biomedical perspective. Finally, it is reinforced that management support is essential for the NC implementation as well as the application of conduct mode technologies to guide professional action. It is recommended that future research explore the manual applicability in different contexts of nursing practice, investigating its effectiveness and challenges in PHC. Furthermore, it is suggested that strategies be developed for its wide dissemination and adherence by professionals, contributing to the qualification of care and expansion of the use of health technologies.

## Data Availability

The research data are available within the article.
