# Supplementary material for: Manual for nursing consultation with people living with Diabetes Mellitus: implementation science
Source: Rev Bras Enferm. 2025 Dec 8;78(Suppl 4):e20240179. doi: 10.1590/0034-7167-2024-0179 (PMC12700573; doi:10.1590/0034-7167-2024-0179)
Supplement: Translation

# Translation: Manual para consulta de enfermagem às pessoas que convivem com Diabetes *Mellitus:* ciência da implementação

**DOI:** 10.1590/0034-7167-2024-0179pt

**Authors:** Simone Orth, Leila Zanatta, Elisangela Argenta Zanatta, Vilma Beltrame, Elizabeth Teixeira, Edlamar Kátia Adamy

**Affiliations:** I Universidade do Estado de Santa Catarina. Chapecó, Santa Catarina, Brasil; II Universidade do Oeste de Santa Catarina. Joaçaba, Santa Catarina, Brasil; III Universidade Estadual do Pará. Belém, Pará, Brasil

**Keywords:** Diabetes *Mellitus*, Consulta de Enfermagem, Tecnologia, Estudo de Validação, Ciência da Implementação.

## Abstract

**Objetivos:**

descrever o processo de validação de conteúdo, implementação e avaliação de uso de um manual para consulta de enfermagem às pessoas que convivem com Diabetes *Mellitus.*

**Métodos:**

pesquisa metodológica, com interface com os princípios da ciência da implementação desenvolvida em três etapas: validação de conteúdo; implementação; e avaliação de uso.

**Resultados:**

conteúdo foi avaliado por sete especialistas, obtendo Índice de Validade de Conteúdo Geral de 0,80 e *Content Validity Ratio* de 0,72. O manual, após ajustes com base em recomendações recebidas, foi intitulado “Consulta do Enfermeiro às pessoas que convivem com Diabetes *Mellitus*”, organizado em 67 páginas, contendo cinco capítulos. A implementação ocorreu em 24 municípios do oeste de Santa Catarina durante quatro meses, e participaram 43 enfermeiros. Avaliação de experiência de uso foi operacionalizada com seis enfermeiros, e da análise, estruturaram-se três categorias.

**Considerações Finais:**

o manual atingiu parâmetros propostos de avaliação, revelando evidências e apropriando-o para uso.

## INTRODUÇÃO

O Diabetes *Mellitus* (DM) é uma doença crônica na qual o corpo não produz insulina ou não consegue empregar adequadamente a insulina que produz. Classificado como uma doença crônica não transmissível, prevalente em todo mundo, é considerado uma condição sensível à Atenção Primária à Saúde (APS)^(1)^.

No ano de 2021, cerca de 536 milhões de pessoas conviviam com o DM no mundo, e a projeção para 2045 é de 783 milhões, um acréscimo de aproximadamente 46%. Comparando a América do Sul e a América Central, em 2021, havia cerca de 32 milhões de indivíduos acometidos pela patologia, com projeção para 2045 de aproximadamente 49 milhões, aumento de 53%. Enquanto isso, o Brasil, em 2021, registrava 15 milhões de indivíduos que conviviam com DM, com uma expectativa para 2045 de aumentar em 47%, equivalendo a aproximadamente 23 milhões de habitantes acometidos pela patologia^(2)^.

Os profissionais que atuam na APS possuem como papel fundamental melhorar tais indicadores; para isso, precisam sensibilizar e auxiliar a população no processo de reconhecimento da patologia, adotando atitudes positivas na prevenção de complicações^(3)^. A criação de vínculo, a análise do perfil da população adscrita em seu território, o manejo dos sintomas e complicações, e o planejamento conjunto entre profissional de saúde, paciente e conjuntura familiar são estratégias essenciais. Logo, o alicerce e o apoio à pessoa que convive com DM permitem um maior controle sobre a patologia e seus efeitos a longo prazo^(4)^.

Assim, com o crescente aumento da prevalência do DM e suas complicações associadas, especialmente no contexto brasileiro, a patologia afeta milhões de indivíduos e está projetada para continuar a crescer nas próximas décadas^(2)^. O DM representa uma carga significativa para o sistema de saúde, sendo uma das principais causas de morbidade e mortalidade no país e no mundo, com impacto direto na qualidade de vida dos indivíduos afetados. Nesse sentido, a APS desempenha um papel fundamental na prevenção e manejo da doença, especialmente quando combinada com a capacidade do enfermeiro em fornecer cuidados adequados, promover o autocuidado e gerenciar as complicações^(3)^.

Nesse contexto, o enfermeiro possui capacidade para proporcionar a melhoria da qualidade de vida dessa população e enfrentamento da doença por meio da consulta de enfermagem (CE). Conforme a Resolução n.º 736 de 2024 do Conselho Federal de Enfermagem (COFEN)^(5)^, a consulta deve ser organizada e registrada conforme as etapas do processo de enfermagem (PE), podendo ser mediada pela aplicação de tecnologias validadas.

A avaliação e implementação dessas tecnologias são realizadas pela ciência da implementação, que associa práticas que são fundamentadas em evidências, levando em consideração fatores como o cenário, as características da população e as características de implementação que podem influenciar a implementação e manutenção delas no contexto em que estão inseridas^(6)^.

Logo, o tempo e a situação financeira são condições essenciais para um projeto ser desenvolvido em sua integralidade por um pesquisador. Além da complexidade de cada fase, o cronograma é, por vezes, o maior adversário do processo. Destarte, é importante a adoção de projetos de continuidade de pesquisa, no intuito de contemplar todas as fases, garantindo que as soluções tecnológicas sejam ativamente implementadas e avaliadas^(7)^.

O manual para a consulta do enfermeiro às pessoas que convivem com DM foi desenvolvido por um estudante de graduação em enfermagem, bolsista de iniciação científica de uma universidade pública localizada no oeste catarinense, em duas fases: na primeira, foi realizada revisão narrativa da literatura; e na segunda, ocorreu a construção. Este artigo diz respeito a estudo de continuidade que validou e avaliou a experiência de uso deste manual, considerado uma tecnologia do tipo material didático instrucional. O manual desenvolvido pôde otimizar a abordagem do profissional na APS, melhorando a adesão ao tratamento, o controle glicêmico e a qualidade de vida dos pacientes que convivem com DM. Ao integrar práticas baseadas em evidências e tecnologias validadas no cuidado de enfermagem, será possível reduzir complicações e promover melhores resultados de saúde para essa população.

Dessa forma, para assegurar a qualidade e aplicabilidade do manual, foi realizada a validação de conteúdo com a participação de especialistas na área, garantindo sua relevância e adequação. A implementação seguiu os princípios do *Consolidated Framework for Implementation Research* (CFIR), permitindo uma abordagem estruturada para sua aplicação prática. Além disso, a avaliação de uso foi conduzida para verificar a efetividade e aceitação do material. Os dados foram analisados por meio do Índice de Validade de Conteúdo (IVC), do *Content Validity Ratio* (CVR), do coeficiente Kappa para concordância entre os avaliadores e da análise de conteúdo de Bardin, assegurando rigor metodológico e confiabilidade dos resultados.

## OBJETIVOS

Descrever o processo de validação de conteúdo, implementação e avaliação de uso de um manual para CE às pessoas que convivem com DM.

## MÉTODOS

### Aspectos éticos

O presente estudo está vinculado ao macroprojeto de pesquisa intitulado “Desenvolvimento de tecnologias para a Consulta do Enfermeiro nas Redes de Atenção à Saúde”, que foi aprovado pelo Comitê de Ética em Pesquisa da Universidade do Estado de Santa Catarina, sob Protocolo n^o^. 5.047.628 e Certificado de Apresentação para Apreciação Ética n^o^. 50165621.2.0000.0118.

### Desenho, período e local do estudo

Trata-se de pesquisa metodológica^(8)^ de caráter exploratório e descritivo, com interface com princípios da ciência da implementação^(9)^, desenvolvida em três etapas adaptadas: validação de conteúdo; implementação; e avaliação de uso. Foi realizada no período de janeiro a dezembro de 2023, caracterizando-se como projeto de continuidade^(7)^.

### Amostra; critérios de inclusão e exclusão

Na primeira fase do estudo, formulou-se uma lista de potenciais especialistas para validação de conteúdo, identificados a partir dos *Curriculum Lattes* registrados na Plataforma do Conselho Nacional de Desenvolvimento Científico e Tecnológico, que contemplassem pelo menos dois dos seguintes critérios de inclusão^(10)^: ser especialista em DM; possuir prática clínico-assistencial com o público-alvo do estudo há, pelo menos, três anos; possuir trabalho publicado em revista e/ou evento sobre DM; ter trabalhos publicados em revistas e/ou eventos sobre construção e validação de tecnologias cuidativo-educacionais em DM; ser membro de Sociedade Brasileira de Diabetes. Os critérios de exclusão foram enviar as respostas fora do prazo determinado.

Para as etapas de implementação e validação, foram estabelecidos os seguintes critérios de inclusão: ser enfermeiro e atuar na APS; atender pessoas que convivem com DM; atuar na região Oeste de Santa Catarina. Os critérios de exclusão foram estar de férias ou licença ou não responder ao questionário dentro do prazo estabelecido.

### Protocolo de estudo

Na primeira fase, foram considerados de seis a 20 especialistas. Recomenda-se^(11)^ um mínimo de seis especialistas, para assegurar um consenso significativo, e no máximo até 20 especialistas, o que contribui para a diversidade de opiniões e redução de vieses individuais, aumentando a robustez dos achados. A validação segundo o modelo confere ao produto uma maior credibilidade, pautada em evidências científicas, ressaltando-se a importância da participação de *experts* no assunto^(12,13)^.

Após o aceite, foram encaminhados o Termo de Consentimento Livre e Esclarecido e as orientações para o preenchimento do instrumento para validação de conteúdo, contendo opção de sugerir melhorias em cada item avaliado, bem como o manual como um todo. O manual foi enviado via e-mail, no mês de janeiro de 2023, com *link* de acesso ao instrumento via *Google Forms* ^®^. Os especialistas tiveram um prazo de 30 dias para devolutiva, porém, devido ao período de férias, o prazo foi estendido por mais 30 dias, finalizando essa etapa do estudo em março de 2023.

O instrumento de validação de conteúdo foi adaptado^(14)^ e organizado em três blocos (1) caracterização dos especialistas; 2) instruções sobre o preenchimento do instrumento; 3) questões para validação de conteúdo), que avaliam os objetivos, a estrutura e a apresentação/relevância do manual, com atribuição de nota em cada item avaliado, conforme o grau de concordância.

No terceiro bloco, o item objetivos foi composto por cinco subitens para análise, sendo 12 voltados à estrutura/apresentação, e cinco, à relevância, totalizando 22 subitens analisados. Cada subitem foi avaliado considerando a escala Likert, atribuindo notas de 1 a 4, sendo: 1 - inadequado; 2 - parcialmente adequado; 3 - adequado; e 4 - totalmente adequado. Quando atribuído grau “1” ou “2”, o instrumento apresentou espaço para a descrição do motivo ou sugestão relacionada ao item avaliado.

Na segunda fase, à luz da ciência da implementação, utilizaram-se os princípios do CFIR, com vistas a implementar e avaliar a experiência de uso. A ciência da implementação permite organizar estratégias de implementação do processo de inserção de medidas e demandas dentro do cenário almejado, permitindo uma análise crítica e integral de todo o processo^(15)^. Utilizaram-se os seguintes domínios do CFIR: características da intervenção; cenário interno; e características dos indivíduos^(9)^. Foi realizada com enfermeiros assistenciais que atuam na APS dos 24 municípios que compõem a Regional de Saúde Oeste de Santa Catarina, que possui 174 enfermeiros atuantes na APS^(16)^. Nessa fase, foram realizados três momentos de divulgação e convite: 1) encaminhamento de e-mail para a gestão da Secretaria de Estado da Saúde e para o enfermeiro responsável técnico de cada município de maio a julho de 2023; 2) realização de encontro *online* com enfermeiros dos municípios no dia 06 de julho de 2023, com duração de 30 minutos; 3) realização de encontro presencial na gerência de saúde da Regional de Saúde Oeste de Santa Catarina, em 17 de agosto, do mesmo ano. Para os municípios que não estiveram representados em nenhuma das ocasiões, foi estabelecido contato via *e-mail* de maio a agosto de 2023. Aceitaram participar 43 enfermeiros da região.

Na terceira fase, após quatro meses de implementação do manual, em dezembro de 2023, foi encaminhado aos mesmos 43 enfermeiros que aceitaram participar do estudo um instrumento para avaliar a experiência de uso. Assim, os enfermeiros receberam um instrumento composto por 16 perguntas abertas, com o objetivo de explorar como o manual foi percebido por eles em relação aos diferentes domínios, considerando aspectos como aceitação, viabilidade e aplicabilidade da tecnologia no cuidado de pacientes que convivem com DM. As perguntas foram formuladas para coletar dados qualitativos que permitissem entender a experiência dos enfermeiros na prática diária, o que foi facilitado pelo uso do *Google Forms* ^®^ como plataforma de coleta de dados. Os enfermeiros tiveram um prazo de 30 dias para responder ao questionário, o que proporcionou tempo suficiente para reflexão e detalhamento das respostas. No uso do CFIR, o planejamento e a implementação das abordagens aos participantes seguem construtos, mas é permitida a escolha que melhor atenda às características e necessidades do estudo, e o uso de todos os construtos não é obrigatório^(9)^.

### Análise dos dados e estatística

Para a validação de conteúdo, os resultados da avaliação dos especialistas foram organizados no programa *Microsoft Office Excel* 2013^®^ e analisados de acordo com a estatística descritiva. com vistas a obter o IVC, que permite a mensuração da proporção de especialistas que estão em concordância com os dados apresentados no manual, permitindo a validação como um todo ou de cada item individualmente. Para o manual ser considerado validado, considerou-se um IVC geral maior ou igual a 0,80. Para o cálculo do IVC, foi utilizada a seguinte fórmula: “IVC = N.º respostas '3'e '4' ÷ N.º total de respostas”^(13)^. Ainda, os dados foram avaliados conforme o CVR, que consiste em uma medida que avalia a validade de conteúdo com base na opinião de especialistas, que classificam os itens como essenciais, úteis ou não essenciais. O CVR é calculado pela fórmula CVR=[2N-(n_e_/2N)] / (N/2), onde n_e_ é o número de especialistas que consideram o item essencial e N é o total de especialistas. O valor do CVR varia de -1 a 1, sendo que valores superiores a 0,59 indicam boa validade de conteúdo. Valores positivos significam que o item é considerado essencial pelos especialistas^(17)^. Os dados foram analisados pelo coeficiente Kappa (*k*), recomendado na análise de dados na área da pesquisa em saúde, com o objetivo em mensurar o nível de concordância entre os especialistas. Isto é, avaliaram-se o grau de concordância, a confiabilidade e a precisão na classificação^(18)^.

Para a avaliação da experiência de uso, as respostas dos participantes foram transcritas na íntegra, utilizando-se a análise de conteúdo categorial, operacionalizada nas fases de pré-análise, categorização, e tratamento dos resultados e interpretação^(19)^.

## RESULTADOS

Participaram da primeira fase (validação de conteúdo) sete especialistas. No que tange à caracterização, verificou-se que: quanto à idade, possuíam entre 35 e 53 anos; quanto ao sexo, cinco eram do sexo feminino e dois eram do sexo masculino; quanto à área de formação, todos eram da enfermagem, com titulação de doutor e tempo de formação de 16 a 32 anos. Quanto à avaliação do manual, foi realizada em uma única rodada, obtendo-se IVC por iten e IVC geral (Tabela 1). Quanto à análise pelo coeficiente *k*, em que se verificou a concordância entre as respostas dos especialistas, obteve-se um valor estatisticamente significativo (*p-value* = 0,0014).

**Tabela 1:** Validação de conteúdo com especialistas, Chapecó, Santa Catarina, Brasil, 2023

Item	TA	A	PA	I	IVC	CVR
**OBJETIVOS**						
1. As informações/conteúdos estão coerentes com as necessidades cotidianas do enfermeiro que atende pessoas que convivem com DM.	2	4	1	0	0,86	0,76
2. O conteúdo do manual permite a compreensão do enfermeiro acerca do DM.	3	3	1	0	0,86	0,76
3. O conteúdo convida e/ou instiga a mudanças de comportamento e atitude do enfermeiro.	2	2	2	1	0,57	0,14
4. O conteúdo incentiva a utilização do manual na prática/atuação.	2	3	1	1	0,71	0,43
5. O conteúdo do manual contribui para esclarecer possíveis dúvidas sobre o DM.	2	3	2	0	0,71	0,43
**ESTRUTRA/APRESENTAÇÃO**						
6. O conteúdo está apresentado com linguagem adequada ao profissional enfermeiro.	4	2	1	0	0,86	0,76
7. O conteúdo apresenta linguagem interativa, capaz de prender a atenção do enfermeiro.	3	3	1	0	0,86	0,76
8. O conteúdo obedece a uma sequência lógica.	4	2	1	0	0,86	0,76
9. O conteúdo do manual contempla as informações pertinentes às etapas da consulta do enfermeiro.	5	1	1	0	0,86	0,76
10. O manual é apropriado para orientar o raciocínio clínico e crítico do enfermeiro.	2	4	1	0	0,86	0,76
11. O conteúdo sobre as etapas da consulta do enfermeiro contempla todas as informações pertinentes.	4	1	2	0	0,71	0,43
12. As informações apresentadas possuem cientificidade.	4	0	3	0	0,57	0,14
13. As informações estão bem estruturadas em concordância e ortografia.	2	3	2	0	0,71	0,43
14. As informações no manual são objetivas e claras.	2	3	2	0	0,71	0,43
15. As informações são esclarecedoras.	2	4	1	0	0,86	0,76
16. As informações são necessárias e pertinentes.	5	2	0	0	1,00	1,00
17. O tamanho e fonte do texto no manual estão adequados.	3	3	1	0	0,86	0,76
**RELEVÂNCIA**						
18. O conteúdo do manual estimula o aprendizado.	4	3	0	0	1,00	1,00
19. O manual contribui para o conhecimento na área.	5	2	0	0	1,00	1,00
20. O conteúdo do manual desperta interesse pela temática.	4	2	1	0	0,86	0,76
21. O manual aborda os assuntos necessários para o saber dos enfermeiros.	5	1	1	0	0,86	0,76
22. O manual está adequado para ser usada pelo enfermeiro na consulta com o paciente que convive com DM.	2	2	2	1	0,57	0,14
Total					0,80	0,72

*DM - Diabetes Mellitus; TA - Totalmente Adequado; A - Adequado; PA - Parcialmente Adequado; I - Inadequado; IVC - Índice de Validade de Conteúdo; CVR - Content Validity Ratio.*

Quanto às sugestões recebidas, foram acatadas 11 das recomendações de ajustes e modificações (Quadro 1), dando-se ênfase, na revisão do manual, aos itens que não obtiveram IVC igual ou maior que 0,80 e/ou CVR igual ou maior que 0,59.

**Quadro 1:** Comentários/sugestões dos especialistas, Chapecó, Santa Catarina, Brasil, 2023

Item	Comentários/sugestões dos especialistas	Julgamento
1	Rever sobre os medicamentos e usar a proposta do Ministério da Saúde em relação aos medicamentos disponíveis na rede SUS.	Acatada.
2	Manual muito extenso para uso do enfermeiro no cotidiano.	Não acatada. Um manual caracteriza-se como um material de comunicação eficaz que reúne de forma sistematizada e organizada informações e dados importantes sobre o assunto, com o intuito de agregar um maior número de pessoas, apresentando conhecimentos essenciais.
3	Indicar a leitura complementar de outros materiais já publicados.	Acatada.
4	Acrescentar o estado hiperglicêmico hiperosmolar.	Acatada.
5	Incluir procedimentos (como a cirurgia bariátrica) para tratar a doença.	Não acatada, uma vez que não era o foco do estudo, além de que o Sistema Único de Saúde dispõe de cirurgia apenas para tratamento de obesidade, conforme Portaria do Ministério da Saúde n.º 424/2013.
6	Reordenação de numerações e imagens.	Acatada.
7	Reordenação dos conteúdos.	Acatada.
8	Substituir a palavra “resumo” por “apresentação”.	Acatada.
9	Usar o avatar em um número maior de páginas.	Acatada.
10	Configurações no instrumento de coleta de dados.	Acatada.
11	Recomendo delinear os cuidados que o cliente deve proceder em caso de hipoglicemia e quais valores são considerados baixos.	Acatada.
12	Recomendo mencionar os sete comportamentos de autocuidado em diabetes da *Association of Diabetes Care & Education Specialists.*	Acatada.
13	Usar citações ao longo do texto de diretrizes atualizadas e informações de organizações que estão diretamente relacionadas ao cuidado em diabetes.	Acatada.
14	Inclusão de um capítulo esclarecendo como o manual foi construído.	Não acatada. O processo metodológico para confecção do manual está descrito em um Trabalho de Conclusão de Curso disponível na biblioteca virtual da universidade.
15	Seguir o consenso internacional quanto às úlceras de pés e pernas e seu rastreamento.	Não acatada. Sociedades especialistas na área fazem uso do termo pé diabético, tal como a Sociedade Brasileira de Diabetes em seu site, sendo que a mesma descreve sobre avaliações e cuidados com o pé diabético.

O manual foi intitulado “Consulta do Enfermeiro às pessoas que convivem com Diabetes *Mellitus*”. Está organizado em 67 páginas e cinco capítulos: definições e explicações sobre o DM; classificações; tratamento não medicamentoso e medicamentoso; principais complicações decorrentes; operacionalização da consulta do enfermeiro (Figura 1). O manual foi registrado com *International Standard Book Number* pela Câmara Brasileira do Livro. Está disponível na íntegra e de forma gratuita na plataforma do *Cofenplay*.

**Figura 1:** Capa do manual “Consulta do Enfermeiro às pessoas que convivem com Diabetes *Mellitus*”, Chapecó, Santa Catarina, Brasil, 2023

Participaram da segunda fase 43 enfermeiros que atuam em 24 municípios. Na terceira fase, participaram seis enfermeiros, sendo de municípios distintos, que devolveram o instrumento encaminhado. A partir da análise e dos construtos adotados, estruturaram-se três categorias: 1 - Características da intervenção (os ajustes ao cenário que foram implementados); 2 - Cenário interno (envolve contexto estrutural, político e cultural); 3 - Características dos indivíduos (o poder de escolha que influencia decisões)^(9)^.

### Características da intervenção

O manual contribui com a execução do PE, orientando a realização das etapas desse processo. A sua organização lógica e sequencial foi destacada por facilitar aos enfermeiros a identificação dos diagnósticos e intervenções de enfermagem.

*Instrumento interessante, simples, fácil de manusear, didático, ajudou muito a montar um instrumento de consulta.* (E1)

*Ele é muito bem-organizado, numa sequência lógica, e nos ajuda a fazer os diagnósticos e intervenções.* (E6)

O manual foi descrito como interessante, simples e fácil de manusear, sugerindo acessibilidade aos enfermeiros. Sua praticidade e relevância das informações valorizam seu uso. Portanto, o manual é uma ferramenta valiosa que combina facilidade de uso, relevância prática e suporte eficaz para executar o PE.

A importância de um manual com evidências teóricas e científicas para sistematizar a consulta é inegável, pois ele serve como um guia didático para executar o PE, fornecendo uma base sólida para tomada de decisão clínica. Ainda, respalda o enfermeiro técnica e cientificamente, garantindo segurança e confiança no desempenho das funções.

*Mais atenção ao exame físico geral e planejamento de ações estratégicas para a prevenção de agravos pela doença.* (E2)

*Ter um respaldo teórico auxilia bastante no processo da consulta* [...] *o manual é didático e exemplifica bem o processo de enfermagem.* (E4)

As afirmações apresentadas destacam a eficácia e a conveniência do manual apresentado de forma digital. Emergiram elogios quanto à sua qualidade, sugerindo apenas atualizações periódicas para acompanhar as mudanças, o que é uma prática comum e necessária na era digital. A facilidade de acesso e uso do manual é enfatizada, reforçando a importância da acessibilidade na experiência de uso do enfermeiro.

*Por ser uma ferramenta digital facilita já que temos acesso ao computador e internet.* (E1)

*O manual é muito bom. Acredito que não mudaria nada, fica na área de trabalho do meu computador, fácil de usar a qualquer momento.* [...] *a princípio, estou usando-o da forma que está.* (E4)

Os enfermeiros destacam a facilidade proporcionada pela ferramenta digital, o que sugere o atendimento de suas necessidades e expectativas.

### Cenário interno

As barreiras para o cuidado adequado aos pacientes com DM são multifacetadas e complexas. Uma barreira significativa é a preferência do paciente por se consultar apenas com o médico da equipe, devido à prescrição medicamentosa. Isso pode limitar o paciente ao acesso de orientações e cuidados adequados.

*Especialmente o tempo, o paciente sempre está com pressa e a demanda da unidade é grande.* (E4)

*Número de profissionais atuantes que torna o dia a dia movimentado, por vezes dificultando a utilização do manual de forma regular. Falta de alguns equipamentos para testes e demais orientações propostas.* (E5)

Outra barreira é a falta de profissionais de apoio, como nutricionistas e educadores físicos, que poderiam fornecer orientações valiosas para os que convivem com DM. Além disso, a demanda elevada de atendimento diário do enfermeiro e o tempo limitado são obstáculos significativos. Os pacientes frequentemente estão com pressa, e a unidade de saúde tem uma grande demanda, o que dificulta a realização de avaliações completas e a utilização regular do manual.

O manual é reconhecido como um recurso informativo que promove a reflexão sobre os cuidados prestados, orientando o planejamento das ações do enfermeiro. Contudo, é valorizado por sua capacidade de ampliar a visão do enfermeiro sobre o atendimento ao paciente, incentivando uma avaliação mais holística que vai além dos indicadores clínicos e considera o contexto familiar do paciente.

*O manual traz informações que nos fazem refletir sobre os cuidados prestados e também orientados às pessoas com DM, consequentemente modificando nossas atitudes em relação ao planejamento das ações. Praticidade, aprimoramento da operacionalização da consulta de enfermagem.* (E2)

*Ele permitiu abrir a visão para atendimento ao paciente, que não é apenas o nível da hemoglobina glicada que tenho que avaliar, mas todo o ser e sua conjuntura familiar. Ele me norteia, não faço mais perguntas vagas.* (E4)

As avaliações destacam a importância do cuidado integral e personalizado ao paciente, enfatizando a necessidade de atenção e cuidados contínuos. A CE é vista como uma ferramenta essencial para melhorar o atendimento ao paciente.

Apesar das dificuldades mencionadas, o enfermeiro experimenta sentimentos de mudanças positivas. O otimismo e a sensação de mudança são evidentes nas declarações a seguir:

[...] *sempre que possível, estar lendo o manual como apoio ao trabalho diário. Estou me adaptando com o manuseio*. [...] *recomendaria o manual para outros colegas enfermeiros. Até já enviei.* (E3)

*Para ter um atendimento padronizado, mas de forma singular. O manual é bem completo e ajuda na tomada de decisão*. (E5)

Destacam a importância e a utilidade do manual para os enfermeiros, recomendando-o aos colegas de profissão e destacando a falta de recursos semelhantes em seu município e a frequência com que atendem esse público específico.

Emergem nas falas a eficácia do manual na padronização do atendimento, ao mesmo tempo que permite uma abordagem singular para cada caso, e a contribuição para a realização do PE e na tomada de decisões. Também enfatizam a importância de se familiarizar com o manual e usá-lo como um recurso de apoio no cotidiano, sugerindo que este não é apenas uma ferramenta útil, mas um material didático instrucional que contribui para qualificar a prática de enfermagem.

### Características dos indivíduos

O manual foi considerado um material didático instrucional valioso que qualifica a CE, considerando sua abrangência, com um conteúdo completo sobre o DM, incluindo estratégias para o atendimento ao paciente e uma seção para entrevista/coleta de dados para a avaliação inicial do paciente.

*É possível executar a consulta de enfermagem com todas as etapas de maneira clara e segura.* (E1)

*Ele atende além do mínimo que devemos fazer por nosso paciente. Às vezes, com a correria do dia a dia, e sem um material para nos guiar na consulta, não realizamos nem o mínimo.* (E6)

Os enfermeiros consideraram a necessidade de outros manuais que abordem demais tipos de atendimento, e destacam a utilidade do manual na prática diária da enfermagem.

Emergem a falta de apoio da Secretaria de Estado da Saúde e a iniciativa individual dos enfermeiros em buscar melhorias para o PE. Embora a gestão apoie as CE de maneira geral, a escolha do material a ser adotado fica a critério do profissional.

*Em meu município, é a iniciativa do próprio enfermeiro para ter melhoras em todo o processo.* (E4)

*Para o uso específico do manual, não, mas a gestão apoia as consultas do enfermeiro de um modo geral, ficando a critério do profissional qual respaldo e material ele prefere utilizar.* (E5)

Isso reforça a ideia de que, habitualmente, são os próprios enfermeiros que fazem o processo de implantação de ações de enfermagem se concretizarem. Sugere a necessidade de maior apoio institucional e recursos para os enfermeiros, a fim de melhorar a eficácia e eficiência do atendimento ao paciente.

## DISCUSSÃO

A realização de trabalho de continuidade permite efetivar a pesquisa em todas as suas etapas, possibilitando que produtos sejam implementados na prática de enfermagem, contribuindo, assim para uma melhoria na práxis, com fundamentação teórica cientifica, e impactos positivos sobre o paciente^(7)^.

A validação de conteúdo permite o aprimoramento do material e qualificação da tecnologia. O manual, em sua versão final, atingiu os parâmetros propostos de avaliação, que comprovaram que o conteúdo é apropriado e válido, sendo possível ser reproduzido no meio científico, pois se obteve um IVC global de 0,80. Tal índice foi semelhante aos obtidos em outros estudos metodológicos de criação de tecnologia com validação de conteúdo que utilizaram IVC para julgamento pelos especialistas, tal como “O Manual de Cuidados de Idosos após Cirurgia Cerebral”, que obteve um IVC geral de 0,80^(20)^, e o trabalho “Construção e Validação de um Manual de Detecção do Pé Diabético para Atenção Primária”, que obteve um IVC geral de 0,85^(21)^.

Quanto à cientificidade, os juízes especialistas na avaliação do conteúdo recomendaram a inclusão de dados adicionais, que foram incorporados no Quadro 1, nos itens 11, 12 e 13. Essas sugestões foram consideradas relevantes e contribuíram para aumentar a fundamentação científica do manual. Vale ressaltar que essas foram as únicas sugestões feitas para aprimorar o aspecto científico do material.

Embora tecnologias do tipo manual possam não ser perceptíveis para o paciente, a utilização destas pelos profissionais se tornou um recurso significativo, visando melhorar a consulta e a comunicação com o paciente. Portanto, elas produzem impactos no setor da saúde, seja na atenção primária ou em outros níveis de atenção^(22)^.

A tecnologia utilizada para a consulta deve ser de fácil entendimento e eficaz, sendo intuitiva para obter melhores resultados. Essas tecnologias surgem como facilitadoras do processo de trabalho do profissional^(23,24)^.

O manual vem ao encontro da legislação do Conselho Federal de Enfermagem^(5)^, que orienta a realização do PE em todos os ambientes que possuam o atendimento de enfermagem. Este processo deve ser sistematizado e organizado de acordo com as necessidades específicas do setor. Tal tipo de tecnologia é inerente à melhoria da CE, proporcionando autonomia ao enfermeiro.

Dessa forma, a autonomia do enfermeiro está intrinsecamente ligada às tecnologias, que servem como suporte técnico e garantia ética em sua prática profissional. Essas tecnologias auxiliam na padronização do atendimento, incentivando a tomada de decisão e o uso de terminologias padronizadas^(25)^. O incentivo ao enfermeiro no uso dessas tecnologias é fundamental para aprimorar a qualidade do cuidado, aliado à segurança e capacitação, o que amplifica a assistência prestada^(26)^.

Embora a CE esteja presente há muitas décadas e seja regulamentada por lei, ainda enfrenta obstáculos para sua efetivação. A prática de realizá-la durante os atendimentos ao paciente é frequentemente negligenciada ou realizada de maneira fragmentada^(27)^. Como mostra o estudo, com a implementação do manual, os enfermeiros estão refletindo sobre a CE e como ela pode melhorar o atendimento ao paciente.

Logo, o manual recebeu *feedback* positivo dos enfermeiros, apontando que a percepção dos profissionais sobre a utilidade, complexidade e adaptabilidade da intervenção pode influenciar a implementação do manual. Estudo que fez o uso de CFIR em um manual de cuidados em saúde apontou que a percepção positiva da intervenção pelos profissionais e a facilidade de uso são razões chave para a adoção e sustentabilidade da intervenção^(28)^.

O enfermeiro entende a relevância do emprego do PE para aprimorar a assistência, mas destaca várias adversidades, incluindo a carga de trabalho excessiva, a escassez de recursos humanos e a falta de treinamentos^(29)^. Existem várias dificuldades mencionadas, incluindo a necessidade de aliviar a carga de trabalho da equipe, com o objetivo de proporcionar tempo e qualidade no atendimento ao indivíduo^(30)^.

Os obstáculos como a carga de trabalho excessiva, a escassez de recursos humanos e a falta de treinamentos que afetam a implementação da CE e o uso do manual estão diretamente relacionados ao domínio do cenário interno no CFIR, que considera como a estrutura e a cultura organizacional afetam a implementação de uma nova intervenção. Em estudo internacional, foi utilizado o CFIR para avaliar a implementação de um manual de cuidados para pacientes com diabetes, e identificou-se que a disponibilidade de recursos humanos e o treinamento contínuo dos profissionais eram fundamentais para a sustentabilidade da intervenção^(31)^.

Logo, o profissional desempenha várias funções, que incluem tanto aspectos gerenciais quanto assistenciais. Ou seja, além de prestar atendimento aos pacientes, eles também organizam a unidade de saúde, supervisionam a equipe e orientam os agentes comunitários de saúde. Portanto, o tempo relatado pelos enfermeiros se torna diversas vezes um empecilho pela grande quantidade de atribuições, e o tempo disponível para o atendimento direto ao paciente acaba se tornando cada vez mais limitado^(32)^. Nesse contexto, o enfermeiro desempenha papel na (re)organização do ambiente de trabalho, enfrentando uma variedade de desafios, seja na dinâmica de trabalho, na gestão de pessoas ou nas necessidades do local. A necessidade de estratégias e adaptações constantes está profundamente ligada ao processo de cuidado^(33)^.

A CE promove a autonomia do enfermeiro, estabelecendo uma conexão com o paciente, e incentiva o planejamento e a organização do trabalho. O enfermeiro é um profissional essencial para o fortalecimento da APS, oferecendo assistência em todas as fases da vida^(34)^.

O desejo de envolver o paciente no cuidado, maximizar o uso do tempo e promover a implementação do PE motiva o enfermeiro a buscar e adotar ferramentas que auxiliem na consulta. Desta forma, ele pode oferecer um atendimento eficaz, padronizado e de alta qualidade^(26)^. Assim, é fundamental o estímulo à autonomia do enfermeiro através do uso de ferramentas de suporte técnico-científico, pois isso promove o empoderamento do profissional diante do cuidado do paciente^(35)^.

Identificar e atender às necessidades genuínas do paciente, evitando intervenções desnecessárias, contribui para reduzir a demanda desnecessária, permitindo que indivíduos com necessidades reais sejam atendidos de maneira tranquila e eficiente. Em outras palavras, a constante reavaliação das ações e suas consequências possibilita um gerenciamento eficaz da demanda real existente na atenção primária^(36)^.

Logo, o binômico CE e as tecnologias no processo de atuação do enfermeiro são pontos importantes no cuidado do paciente, tornando-se inerente ao processo de cuidar. A tecnologia usufruída na CE permite a coleta de dados e seu tratamento coerentemente, resultando em excelente prática do profissional^(23)^.

Outro estudo que utilizou *framework* para avaliar a implementação de intervenções em saúde relatou que, quando as tecnologias e os processos de trabalho são adaptados corretamente e os profissionais se sentem apoiados e capacitados, as intervenções têm um impacto positivo no atendimento ao paciente e nos resultados de saúde. No estudo, o impacto do manual é claramente visível na qualidade do cuidado prestado e no fortalecimento da autonomia do enfermeiro, o que é um reflexo dos resultados positivos apontados pelo CFIR^(37)^.

Impulsionar esses profissionais a usufruir de ferramentas e lhes apresentar retorno notórios em sua prática alimentará a ambição por mais resultados positivos. A necessidade de planejamento conjuntamente com a gestão para incorporar o PE na APS é indispensável para a melhoria no atendimento à população^(29)^.

### Limitações do estudo

As limitações encontradas neste estudo foram o curto prazo entre a implementação das estratégias para o uso do manual na APS e a subsequente avaliação da experiência de uso. Portanto, sugere-se que estudos futuros considerem um período de tempo mais longo para permitir uma avaliação mais precisa das estratégias implementadas.

### Contribuições para as áreas da enfermagem, saúde ou políticas públicas

O manual está disponível para utilização pelos enfermeiros, visando potencializar práticas direcionadas ao atendimento à população que convive com DM na APS e, com isso, fortalecer o processo de trabalho do enfermeiro, contribuindo para a qualidade de vida dessa população. O manual é aplicável em diferentes cenários de ensino, incluindo graduação, pós-graduação, residência e também em atividades de educação contínua ou permanente.

## CONSIDERAÇÕES FINAIS

O manual atingiu os parâmetros propostos de avaliação, revelando evidências que está apropriado para uso durante a CE. O manual permite o acesso rápido e fácil às informações, tanto para uso na APS quanto na formação/capacitação de enfermeiros, e contém ilustrações que facilitam o manuseio e o entendimento do enfermeiro acerca do conteúdo. O uso do manual possibilita segurança nas ações do profissional, mas ressalta-se a necessidade de conquistar o público-alvo com vistas a desmistificar a ideia de que o cuidado de pessoas que convivem com DM se limita à perspectiva clínico-biomédica. Reforça-se, por fim, que o apoio da gestão é fundamental para a implementação da CE, bem como a aplicação de tecnologias de modo de conduta para guiar o agir profissional. Recomenda-se que pesquisas futuras explorem a aplicabilidade do manual em diferentes contextos da prática de enfermagem, investigando sua efetividade e desafios na APS. Além disso, sugere-se o desenvolvimento de estratégias para sua ampla disseminação e adesão pelos profissionais, contribuindo para a qualificação do cuidado e ampliação do uso de tecnologias em saúde.

## Data Availability

Os dados da pesquisa estão disponíveis no corpo do artigo.
